# A survival prediction model and nomogram based on immune-related gene expression in chronic lymphocytic leukemia cells

**DOI:** 10.3389/fmed.2022.1026812

**Published:** 2022-12-19

**Authors:** Han-ying Huang, Yun Wang, Tobias Herold, Robert Peter Gale, Jing-zi Wang, Liang Li, Huan-xin Lin, Yang Liang

**Affiliations:** ^1^State Key Laboratory of Oncology in South China, Collaborative Innovation Center for Cancer Medicine, Sun Yat-sen University Cancer Center, Guangzhou, China; ^2^Department of Hematologic Oncology, Sun Yat-sen University Cancer Center, Guangzhou, China; ^3^Department of Radiation Oncology, Sun Yat-sen University Cancer Center, Guangzhou, China; ^4^Laboratory for Leukemia Diagnostics, Department of Medicine III, University Hospital, LMU Munich, Munich, Germany; ^5^Haematology Research Centre, Department of Immunology and Inflammation, Imperial College London, London, United Kingdom

**Keywords:** chronic lymphocytic leukemia, gene expression, immune, prognosis, model

## Abstract

**Introduction:**

There are many different chronic lymphoblastic leukemia (CLL) survival prediction models and scores. But none provide information on expression of immune-related genes in the CLL cells.

**Methods:**

We interrogated data from the Gene Expression Omnibus database (GEO, GSE22762; Number = 151; training) and International Cancer Genome Consortium database (ICGC, CLLE-ES; Number = 491; validation) to develop an immune risk score (IRS) using Least absolute shrinkage and selection operator (LASSO) Cox regression analyses based on expression of immune-related genes in CLL cells. The accuracy of the predicted nomogram we developed using the IRS, Binet stage, and del(17p) cytogenetic data was subsequently assessed using calibration curves.

**Results:**

A survival model based on expression of 5 immune-related genes was constructed. Areas under the curve (AUC) for 1-year survivals were 0.90 (95% confidence interval, 0.78, 0.99) and 0.75 (0.54, 0.87) in the training and validation datasets, respectively. 5-year survivals of low- and high-risk subjects were 89% (83, 95%) vs. 6% (0, 17%; *p* < 0.001) and 98% (95, 100%) vs. 92% (88, 96%; *p* < 0.001) in two datasets. The IRS was an independent survival predictor of both datasets. A calibration curve showed good performance of the nomogram. *In vitro*, the high expression of CDKN2A and SREBF2 in the bone marrow of patients with CLL was verified by immunohistochemistry analysis (IHC), which were associated with poor prognosis and may play an important role in the complex bone marrow immune environment.

**Conclusion:**

The IRS is an accurate independent survival predictor with a high C-statistic. A combined nomogram had good survival prediction accuracy in calibration curves. These data demonstrate the potential impact of immune related genes on survival in CLL.

## Introduction

There are several survival prediction models and scores for chronic lymphocytic leukemia (CLL) including Binet and Rai staging systems, MD Anderson Cancer Center 2007 index score, CLL International Prognostic Index (CLL-IPI), Barcelona-Brno score, International Prognostic Score-A (IPS-A), CLL-01 and a tailored approach (1–5). These models and scores include clinical, laboratory and genetic co-variates such as age, disease stage, Eastern Cooperative Oncology Group (ECOG) performance score, immunoglobulin variable heavy chain (*IGHV*) mutation state, cytogenetic abnormalities, especially del(11) and del(17p), serum β2-microglobulin, lymphocyte doubling time and others. These models and scores have only modest accuracy with C-statistics of 0.60–0.77 (6). Data on the expression of immune-related genes in CLL cells is not incorporated in any survival prediction, prognostic model, or score.

We developed a survival predictive model we termed the Immune Risk Score (IRS) by analyzing immune-related gene expression data in CLL cells from two publicly available datasets. We evaluated the model in multivariable analyses and verified it in a validation dataset. We combined the IRS with Binet stage and del(17p) cytogenetics data to develop a survival prediction nomogram.

## Materials and methods

### Data collection

Data from 2 CLL datasets including 642 subjects with micro-array survival data, GSE22762 and CLLE-ES (7, 8), were obtained from the Gene Expression Omnibus (GEO) database and the International Cancer Genome Consortium (ICGC) database. The Data Access Committee approved access to this data under DACO-7056. Gene expression profiles were derived from the microarray of subjects’ peripheral blood or bone marrow in two datasets and normalized between different arrays by sva package used to remove batch effects in high-throughput experiments to reduce dependence, stabilize error rate estimates and improve repeatability (9, 10). Download the list of immune-related genes from the ImmPort database^1^ for extracting all immune genes in the GEO dataset and the ICGC dataset, to obtain a list of candidate immune genes for further study. The workflow is summarized in Supplementary Figure 1.

### Construction and validation of the IRS

The GSE22762 dataset was used for training to construct the IRS model. Univariable Cox regression analyses were used to identify immune-related genes significantly correlated with survival at *p* < 0.05. We used Least Absolute Shrinkage and Selection Operator (LASSO) Cox regression analyses to identify the best weighting coefficient of immune-related genes for survival. The maximum likelihood estimate of penalty after 1,000-fold cross-validation was determined and the 1-SE criterion was used to determine the optimal value of the penalty parameter λ. These data were used to develop the IRS. The CLLE-ES dataset was used as validation set.

Prediction accuracy of the IRS was quantified using time-dependent receiver-operating characteristic (ROC) curves and the area under the curve (AUC) method. Subjects were divided into high- and low-risk cohorts by the optimal cut-off of risk score in each dataset. Survivals of subjects in the high- and low-risk cohorts were compared by Kaplan–Meier curves with log-rank tests. The GSE22762 and CLLE-ES datasets had clinical co-variates which were included in uni and multivariable Cox regression analyses.

### Nomogram development and validation

We developed a survival prediction nomogram by combining the IRS with Binet stage and del(17p) cytogenetics data. In the nomogram, each co-variate is assigned a point and added to give a total point corresponding to 1-, 3-, or 5-year survival. The calibration curve showed the relationship between the predicted value and the measured value of the nomogram in the training dataset. The IRS, clinical and laboratory co-variates and nomogram were compared with the time-dependent receiver operating characteristic curve (time-ROC curve) for 5-year survival.

### Patient selection and follow-up

We collected 44 newly diagnosed CLL patients, whose diagnosis was confirmed by histopathology following bone marrow aspiration procedures taking place between 2011 and 2020 at Sun Yat-sen University Cancer Center (SYSUCC), Guangzhou, China. The other inclusion criteria were: No acute infection 2 weeks prior to enrollment; no chronic inflammation or autoimmune disease; and no consumption of immunosuppressive or immunomodulatory drugs. All patients were followed-up until September 2020 through hospital records or phone contact with the patients or relatives who were aware of their illness. This study was approved by the Research Ethics Committee of SYSUCC (Approved number B2021-044-01) and was conducted in accordance with the Declaration of Helsinki.

### Immunohistochemistry analysis

We verified two immune-related genes (CDKN2A and SREBF2) with the larger weight coefficient according to IRS model, by immunohistochemistry analysis. Several types of immune cells, including monocytes, CD4^+^ T cells, CD8^+^T cells, and neutrophils were selected to further explore the relationship between immune genes and the bone marrow immune environment.

Briefly, the paraffin-embedded bone marrow puncture specimens were stained using antibodies against CD4^+^ T cells (anti-CD4 antibody, ZSGB_BIO, Catalog No. ZA-0519), CD8^+^ T cells (anti-CD8 antibody, ZSGB_BIO, Catalog No. ZA-0508), monocytes (anti-CD14 antibody, SANTA, Catalog No. sc-58951), neutrophils (anti-CD66b antibody, ABCAM, Catalog No. ab197678), and two immune-related genes: the sterol regulatory element binding transcription factor (SREBF2; anti-SREBF2 antibody, ABCAM, Catalog No. ab30682), and the cyclin dependent kinase inhibitor 2A (CDKN2A; anti-CDKN2A antibody, Affinity, Catalog No. AF0228). Every five duplicate slide was analyzed by two independent researchers in a blinded manner. For the immune cells, researchers recorded the number of positive immune cells and calculated the density of immune cells using Image J software (National Institute of Health, Bethesda, MD, USA). For the immune-related genes, we recorded the staining area and degree of immune protein expression using a 1–3-point system. The points were added up to obtain a total immunohistochemistry analysis score for each immune-related gene.

### Statistics

Subjects were divided into high- or low-risk cohorts at the optimal cut-off IRS which was determined by ROC curves analyses. Prediction accuracy was quantified using time-dependent ROCs and AUCs calculated to determine the Concordance C-Statistic. Survival was defined as the interval from diagnosis to death from any cause. Time-to-therapy (TTT) was defined as the interval from diagnosis to start of 1st or next treatment. A significant proportion of patients in GSE22762 were not analyzed at diagnosis but at a more advanced disease stage and in relapse. Survival and TTT in those patients were calculated as the interval from measurement to the respective event. Survivals were compared in Kaplan–Meier curves using the log-rank test. In univariable Cox regression analyses co-variates with *P* < 0.05 were used in subsequent multivariable Cox regression analyses. SPSS software version 24.0 (SPSS, Inc., Chicago, IL, USA), R software version 3.6.2 (R Foundation for Statistical Computing, Vienna, Austria), and Perl version 5.24.3 (Perl Foundation, Holland, MI, USA) were used for statistical analyses. A two-sided *P* < 0.05 was considered significant.

## Results

### Subject selection and IRS development

Clinical and laboratory co-variates of 632 subjects from GSE22762 and CLLE-ES were displayed in Table 1. LASSO Cox regression analysis was used to determine the five genes of model (shown in Supplementary Figure 2A). The optimal weighting coefficient for each gene was determined by the regularization parameter lambda (shown in Supplementary Figure 2B). Five genes with optimal coefficients were selected to develop the IRS, including *CDKN2A*, *SREBF2*, *NRIP1*, *BCL11B*, and *SIRT1* (Supplementary Table 1). The equation used to calculate the IRS is:

**TABLE 1:** Clinical co-variates in the training and validation datasets.

	Training dataset GSE22762 (*N* = 149)	Validation dataset CLLE-ES (*N* = 483)	*P*-value
Female	88 (59%)	191 (40%)	<0.001
Age			
<65 y	89 (60%)	278 (58%)	0.70
≥65 y	60 (40%)	205 (42%)	
Median (y, range)	63 (30–84)	62 (28–87)	
del (6q)	8 (5%)	NA	
del (11q)	18 (12%)	NA	
del (13q)	85 (57%)	NA	
del (17p)	13 (9%)	NA	
+12	20 (13%)	NA	
*TP53*	NA	14/463 (3%)	
*IGHV*. Unmutated	64/132 (48%)	156/458 (32%)	<0.01
Binet stage			<0.001
A	58/105 (39%)	427/480 (88%)	
B	23/105 (15%)	39/480 (8%)	
C	24/105 (16%)	14/480 (3%)	
OS status	110/149 (74%)	416/483 (86%)	<0.01
OSmedian time (y)	3.76 (0.09–7.38)	7.13 (0.06–26.52)	
Median TTT (y)	1.04 (0–6.50)	NA	

*N*, number; y, years old; OS, overall survival; TTT, time to treatment.

IRS = (0.54 × *CDKN2*A RNA expression) + (0.27 × *SREBF2* RNA expression) − (0.04 × *SIRT1* RNA expression) − (0.11 × *BCL11B* RNA expression) − (0.26 × *NRIP1* RNA expression).

We calculated the IRS for each subject in both datasets according to this equation dividing subjects into high- and low-risk cohorts based on the optimal IRS cut-off of the corresponding dataset. The training and validation data sets’ optimal cut-off values are 2.29 and 1.94, respectively.

### IRS validation

**FIGURE 1:**
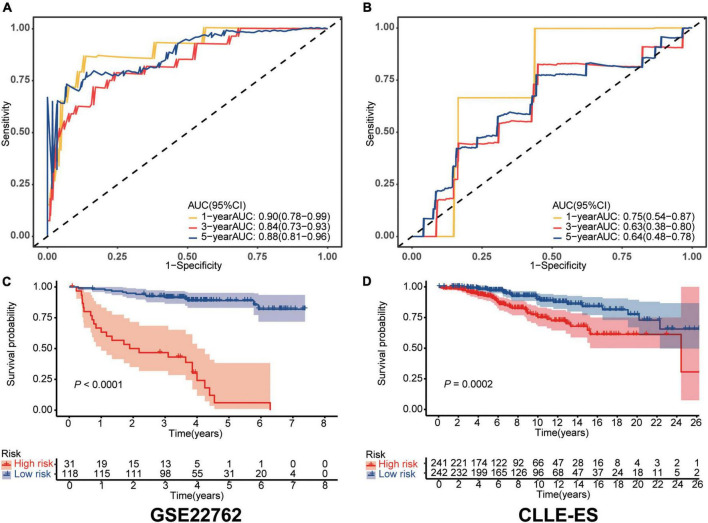
Validation of the immune risk score model. **(A,B)** Sensitivity and specificity of the IRS model were assessed in each dataset by time-dependent ROC analyses. **(C,D)** Survival differences between high- and low-risk cohorts in each dataset.

Sensitivity and specificity of the IRS in each dataset were assessed by time-dependent ROC analyses. AUCs in the training dataset for 1-, 3-, and 5-year survivals were 0.90 [95% Confidence Interval, (CI), 0.78, 0.99), 0.84 (0.73, 0.93), and 0.88 (0.81, 0.96; shown in Figure 1A]. Comparable data for the validation dataset were 0.75 (0.54, 0.87), 0.63 (0.38, 0.80), and 0.64 (0.48, 0.78; shown in Figure 1B). We also compared survival of the high- and low-risk cohorts using Kaplan–Meier curves in each dataset (shown in Figures 1C, D). In the training dataset, subjects were divided into high- and low-risk groups using the optimal cut-off value of IRS. The high- and low-risk groups had 5-year survival of 89% (83, 95%) vs. 6% (0, 17%; *P* < 0.0001). Comparable survivals in the validation dataset were 98% (95, 100%) vs. 92% (88, 96%; *P* < 0.001). The IRS in each dataset was an independent prognostic factor in multivariate Cox regression analysis (Table 2). Moreover, time to treatment (TTT) was statistically different between high-risk and low-risk cohorts in the training dataset (*P* < 0.001, shown in Supplementary Figure 3). In addition, the 64 patients who were initially diagnosed with CLL in the training set were divided into subgroups to further verify the difference in survival between high and low immune score groups, as well as the sensitivity and specificity for predicting survival, all of which were well validated (shown in Supplementary Figure 4).

**TABLE 2:** Univariable and multivariable regression analyses of survival.

Characteristics	Uni-variable		Multi-variable	
	Hazard ratio (95% CI)	*P-*value	Hazard ratio (95% CI)	*P-*value
**Training cohort** **GSE22762 (*N* = 149)**				
Age (< vs. ≥ 65 y)	1.00 (0.53, 1.89)	0.99		
Sex (F/M)	1.38 (0.73, 2.58)	0.32		
del (6q) (N/Y)	3.75 (1.57, 8.99)	<0.01	0.84 (0.26, 2.75)	0.77
del (11q) (N/Y)	2.35 (1.07, 5.13)	0.03	3.35 (0.84, 13.33)	0.09
del (13q) (N/Y)	1.05 (0.56, 2.00)	0.87		
del (17p) (N/Y)	8.09 (3.88, 16.85)	<0.001	19.71 (5.17, 75.12)	<0.001
+ 12 (N/Y)	1.45 (0.64, 3.29)	0.38		
*IGHV* mutated (N/Y)	0.26 (0.12, 0.55)	<0.001	0.53 (0.19, 1.51)	0.24
Binet stage (A *vs.* B vs. C)	2.53 (1.57, 4.10)	<0.001	2.18 (1.18, 4.02)	0.01
Immune risk score	20.8 (9.4, 45.9)	<0.001	5.41 (1.69, 17.28)	<0.01
**Validation cohort** **CLL-ES (*N* = 483)**				
Age (< vs. ≥ 65 y)	1.99 (1.22, 3.23)	<0.01	1.00 (0.60, 1.66)	0.99
Sex (F/M)	1.53 (0.92, 2.57)	0.11		
*TP53* (N/Y)	1.49 (0.36, 6.10)	0.58		
*IGHV* mutated (N/Y)	0.12 (0.07, 0.22)	<0.001	0.72 (0.40, 1.29)	0.27
Binet stage (A vs. B vs. C)	1.65 (1.08, 2.53)	0.02	0.23 (0.03, 1.51)	0.13
Immune risk score	2.63 (1.30, 5.32)	<0.01	2.61 (1.24, 5.48)	0.01

CI, confidence interval; *N*, number; y, years old; F, female; M, male; N/Y, no/yes.

### Nomogram

**FIGURE 2:**
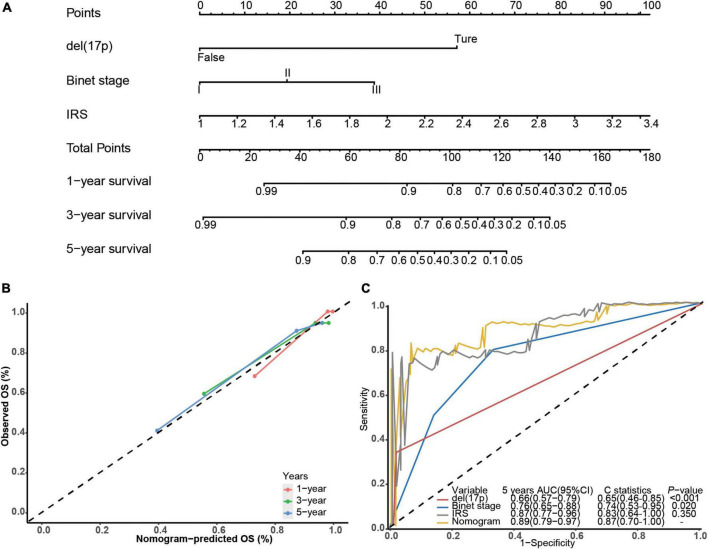
Building the survival nomogram. **(A)** The combined nomogram plot was built in the training dataset. **(B)** Time-dependent ROC curves of training nomogram were compared based on co-variates 1-, 3- and 5-year survivals. **(C)** 5-year ROC curves and C-statistics of the nomogram compared with other co-variates in the training dataset.

Next, we built a survival nomogram for the training dataset combining the IRS with Binet stage and del(17p) data (shown in Figure 2A). (There were too few deaths and lack of data in the validation cohort for nomogram building.) A calibration curve showed good accuracy predicting 1-, 3-, and 5-year survivals (shown in Figure 2B). The nomogram increased 5-year survival prediction accuracy compared with IRS only, Binet stage only and del(17p) only (shown in Figure 2C). The concordance statistics of the nomogram was 0.87 (0.70, 1.00) compared with concordance statistics of 0.83 (0.64, 1.00), 0.74 (0.53, 0.95), and 0.65 (0.46, 0.85), respectively.

### *In vitro* validation in bone marrow of CLL patients by IHC

**FIGURE 3:**
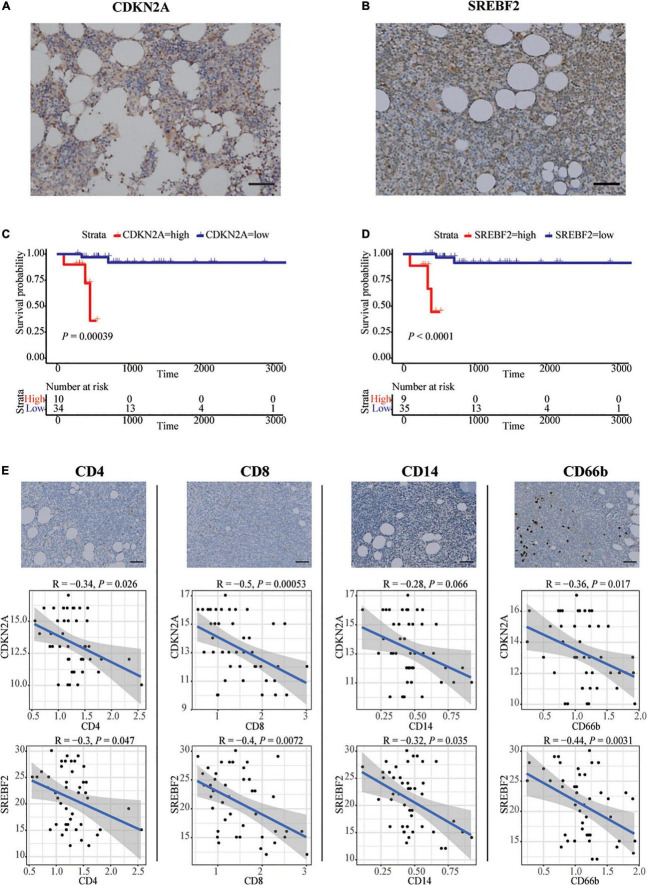
*In vitro* validation in bone marrow of CLL patients by IHC. **(A,B)** Representative images of SREBF2, and CDKN2A -positive cells in a bone marrow sample taken from one CLL patient. Scare bar: 50 μm. **(C,D)** Kaplan–Meier curves for overall survival of CLL patients associated with SREBF2 and CDKN2A expression. **(E)** Representative images of CD4, CD8, CD14, and CD66b -positive cells in a bone marrow sample taken from one CLL patient. Scare bar: 50 μm. Correlation analysis between two immune-related genes and immune cell types (Spearman analysis).

Forty-four patients newly diagnosed with CLL were retrospectively enrolled in our study. The age range was 28–78 years old, and the survival time range was 96–3,371 days. Immunohistochemistry analysis experiments confirmed high expression of SREBF2 and CDKN2A proteins in the bone marrow of CLL patients (Figures 3A, B). In addition, the information on each immunohistochemical indicator was shown in the Table 3. According to the best cut-off value of the immunohistochemistry analysis score, the prognosis of CLL patients in the high expression group was significantly worse than that in the low expression group (*P* < 0.05; Figures 3C, D). Interestingly, SREBF2 expression levels were negatively correlated with the infiltration of CD8^+^ T cells, CD4^+^ T cells, CD14^+^ monocytes, and CD66b^+^ neutrophils, whereas CDKN2A expression negatively correlated with the presence of CD8^+^ T cells, CD4^+^ T cells, and CD66b^+^ neutrophils (*P* < 0.05, Figure 3E).

**TABLE 3:** The mean, the best cut-off value, and the number of cases with high and low IHC scores for each immunohistochemical index.

Index	CD8	CD4	CD14	CD66b	SREBF2	CDKN2A
Mean ± variance	1.48 ± 0.6	1.27 ± 0.4	0.46 ± 0.2	1.12 ± 0.4	18.68 ± 3.8	7.86 ± 2.3
The best cut-off	1.65	1.09	0.44	0.75	15	6
High score (N)	15 (34%)	31 (70%)	22 (50%)	37 (84%)	34 (77%)	31 (70%)
Low score (N)	29 (66%)	13 (30%)	22 (50%)	7 (16%)	10 (23%)	13 (30%)

## Discussion

No CLL survival prediction model includes data on expression of immune-related genes in chronic lymphocytic leukemia cells. Using data on immune-related gene expression from the public GSE22762 dataset, we developed a prognostic model, the IRS, which we independently verified in the CLLE-ES dataset. In our model high expression of *NRIP1, BCL11B* and *SIRT1* were associated with a good and high expression of *CDKN2A* and *SREBF2* with poor prognoses. Functions of these genes are summarized in Supplementary Table 2. Areas under the curve (AUC) for 1-year survivals in the training and validation datasets were 0.90 [95% Confidence Interval (CI), 0.78, 0.99] and 0.75 (0.54, 0.87). With the combination of Binet stage and del(17p) cytogenetic data, we developed a nomogram that enhanced the accuracy of survival prediction, with a C-statistic of 0.87 (0.70, 1.00) in the training set. Due to missing data, we were not able to test the nomogram in the validation set.

*NRIP1*, *BCL11B*, and *SIRT1* are the 3 genes whose elevated expression is linked to better survival. *NRIP1*, expression is associated with a favorable prognosis in previous CLL studies and may operate *via* the NF-κB pathway and/or Wnt signaling pathways in CLL (7, 11). Del(17p13) and del(11q22) are associated with low *NRIP1* expression as unmutated *IGHV* state (12, 13). Consequently, high *NRIP1* expression in CLL may be a surrogate for absence of one or more of adverse prognostic co-variates. Of note, a recent study identified *NRIP1* as a partner of the NRIP1-MIR99AHG fusion in acute myeloid leukemia. Although it is a rare fusion, it is suspected to be associated with poor clinical outcome (14).

*BCL11B* regulates thymocyte development and is a cancer suppressor gene in T-cell leukemia/lymphoma (15–19). *BCL11B* inactivation in normal thymocytes triggers cell proliferation, *TP53*-dependent apoptosis and increases growth of transformed lymphocytes (20). *BCL11B* up-regulates *NKG2D* ligands of the major histocompatibility complex class I-related molecules A and B (MICA and MICB), promotes the anti-tumor response of T- and NK-cells and prevents immune evasion of colon cancer cells (21). Down-regulation of *BCL11B* expression inhibits proliferation and induces apoptosis in malignant T-cells (22, 23). There are no data other than ours on the impact of *BCL11B* expression in CLL.

*SIRT1* is up-regulated in some cancers including acute and chronic myeloid leukemias (AML and CML) but down-regulated in breast and liver cancers (24, 25). Low *SIRT1* expression increases abnormal self-renewal of myelodysplastic syndrome (MDS) stem cells by enabling over-acetylation and reducing the activity of TET2 (26). Inhibition of *SIRT1* expression promotes the growth of T-cell ALL by activating the NOTCH and NF-κB pathways (27). The impact of *SIRT1* expression in other on hematologic cancers is controversial. Two studies reported *SIRT1* is over-expressed in primary CLL cells and in the JVM-3 and MEC-2 CLL cell lines and that inhibiting *SIRT1* expression might activate the p53 pathway inhibiting cell proliferation and promoting apoptosis in CLL (28, 29).

Increased expression of *CDKN2A* and *SREBF2* correlated with worse survival. CDKN2A is a rather contradictory gene. Manifested as inactivation of CDKN2A and p53 in pancreatic cancer (30). However, CDKN2A is highly expressed in gastric precancerous lesions but decreased expression in gastric cancer stage (31). This appears to have similar changes in hematologic malignancies. In this study, CDKN2A was highly expressed in CLL/SLL. CDKN2A is inactivated when CLL is converted to Richter syndrome; CDKN2A inactivation combined with mutations in TP53, MYC and NOTCH1 are associated with transformation of CLL to Richter syndrome (32–34). At present, the expression level of CDKN2A in CLL patients has not been clearly reported. These data represent different meanings of CDKN2A at different stages. *SREBF2* expression is associated with a poor prognosis in T-cell lymphoma, AML, plasma cell myeloma and liver cancer (35–38). There are no data on the impact of *SREBF2* expression in CLL.

According to previous research, the presence of immune cells (CD8^+^ T cells, CD4^+^ T cells, CD14^+^ monocytes, and CD66b^+^ neutrophils) in the bone marrow immune environment correlated positively with good CLL prognosis (39, 40). We hypothesize that upregulation of SREBF2 and CDKN2A may be associated with decreased infiltration of bone marrow immune cells. This may be an underlying mechanism that causes CLL immunosuppression.

Why should immune gene expression in leukemia cells correlate with prognosis? We hypothesize expression of these genes reflects biologic features of the leukemia cells, but it is also possible there is an interaction with the host immune response, cancer micro-environment, both or something else. We also emphasize we are reporting correlations, not necessarily *cause-and-effect.*

Our study has several limitations. 1st, the datasets we interrogated lacked more important clinical and/or laboratory co-variates (such as CLL-IPI score) limiting our multivariable analyses. 2nd, survival of subjects in the validation set was significantly better than in the training dataset reflecting different therapies and disease stage, which has the advantage of allowing us to term the IRS a prognostic rather than predictive score. However, the very good survival of the high- and low-risk cohorts in the validation cohort decreased but did not negate discrimination power of the IRS. 3rd, the large differences between the datasets make it impossible to define a uniform cut-off. Future studies are necessary to establish an optimal cut off.

In conclusion, we developed and validated a prognostic survival model for CLL based on the expression of 5 immune-related genes which had high accuracy. We also developed a survival nomogram by adding Binet stage and del(17p) cytogenetics data to the IRS which improved the C-statistic. Two immune-related genes were predicted and validated using immunohistochemistry analysis experiments, which may play an important role in the complex bone marrow immune environment.

## Data availability statement

The original contributions presented in this study are included in the article/Supplementary material, further inquiries can be directed to the corresponding author/s.

## Ethics statement

This study protocol was reviewed and approved by the Data Access Committee (Approval number DACO-7056) from the ICGC database, without written informed consent. This study was also approved by the Research Ethics Committee of SYSUCC (Approval number B2021-044-01). Research was conducted in accordance with guidelines for human research and was conducted ethically in accordance with the World Medical Association Declaration of Helsinki.

## Author contributions

H-YH wrote the original manuscript. YL, RG, H-YH, and YW reviewed and edited the manuscript. H-YH and YW performed the software analyses. TH, J-ZW, and LL collected the data. YL and H-XL were responsible for the research design, project administration, and funding. All authors reviewed the final manuscript and agreed to submit for publication.

## Footnotes

1
https://www.immport.org/about
