# Supplementary material for: A survival prediction model and nomogram based on immune-related gene expression in chronic lymphocytic leukemia cells
Source: Front Med (Lausanne). 2022 Dec 19;9:1026812. doi: 10.3389/fmed.2022.1026812 (PMC9806429; doi:10.3389/fmed.2022.1026812)
Supplement: Supplementary file 1 [file Data_Sheet_1.pdf]

Supplementary materials

Supplementary Table 1. Five genes selected to construct an immune risk score by LASSO Cox regression analysis.

Gene Name	Coefficient
CDKN2A	0.54
SREBF2	0.27
SIRT1	-0.04
BCL11B	-0.11
NRIP1	-0.26

Supplementary Table 2. The functions of 5 immune-related genes.

Gene name	Functions	References
NRIP1	*M*ay promote progression of B-cell cancers through the NF-*κ*B pathway and/or Wnt signaling including CLL.	7, 11-14
BCL11B	No data in CLL. *R*egulates development of thymocytes and is a cancer suppressor gene. Promotes proliferation of malignant T-cells and inhibits apoptosis.	15-23
SIRT1	Low expression increases abnormal self-renewal of MDS stem cells. Up-regulated in AML, CML and CLL. May promote cell proliferation and inhibit apoptosis of CLL cells by down-regulating the p53 pathway.	24-29
CDKN2A	*I*nactivation combined with mutations in *TP53, MYC* and *NOTCH1* is associated with transformation to a diffuse large B-cell lymphoma.	30-34
SREBF2	No data in CLL. *E*xpression is associated with a poor prognosis in T-cell lymphoma, AML, plasma cell myeloma and liver cancer.	35-38

Figure legends

Supplementary Figure 1. Workflow of prognostic immune gene signatures.

Supplementary Figure 2. Construction of the immune model. (A) 1,000 bootstrap replicates by LASSO Cox regression analysis for variable selection. (B) LASSO coefficients of immune genes. Each curve represents an immune gene.

Supplementary Figure 3. Time to treatment differences between high- and low-risk cohorts in training dataset.

Supplementary Figure 4. Validation of 64 patients initially diagnosed with CLL in the training set. (A) Survival differences between high- and low-risk cohorts in 64 patients. (B-D) Sensitivity and specificity of the IRS model were assessed in 64 patients.
