# Supplementary figures and images for: A survival prediction model and nomogram based on immune-related gene expression in chronic lymphocytic leukemia cells
Source: Front Med (Lausanne). 2022 Dec 19;9:1026812. doi: 10.3389/fmed.2022.1026812 (PMC9806429; doi:10.3389/fmed.2022.1026812)

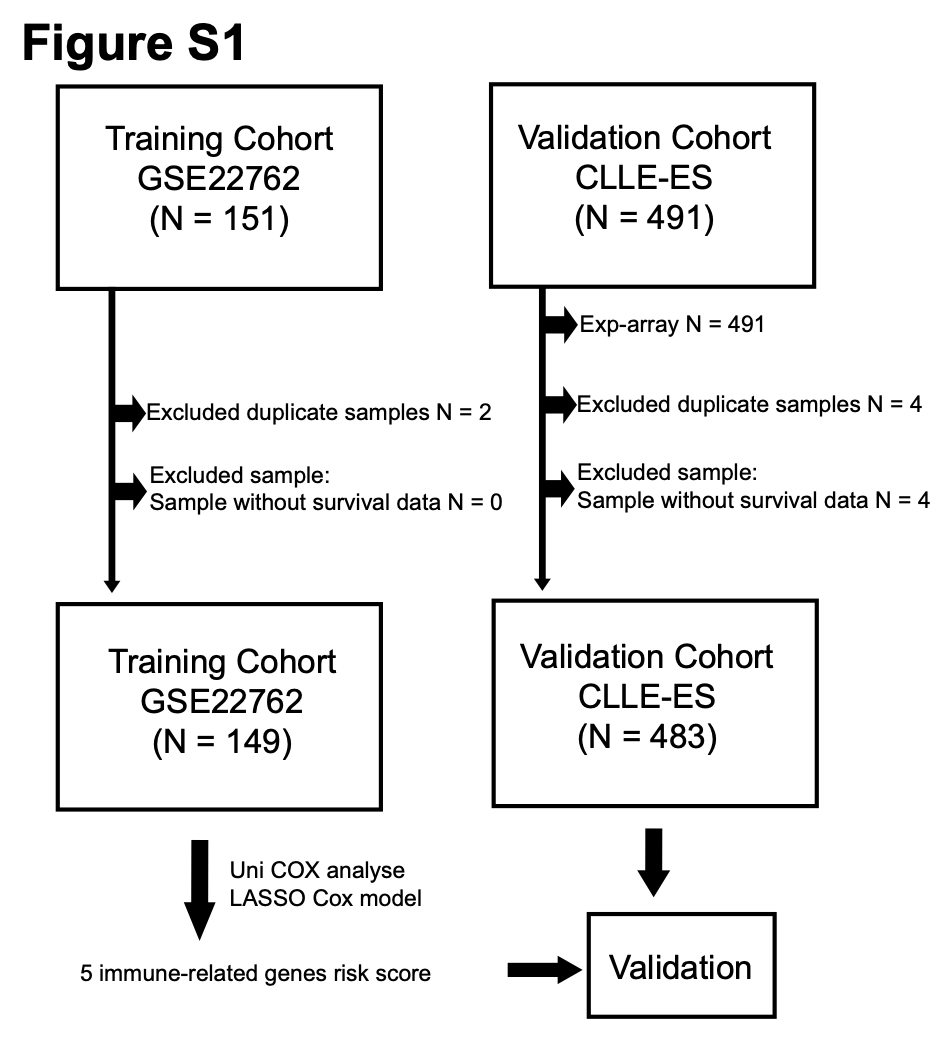

Supplement: Supplementary file 2 [file Image_1.TIFF]

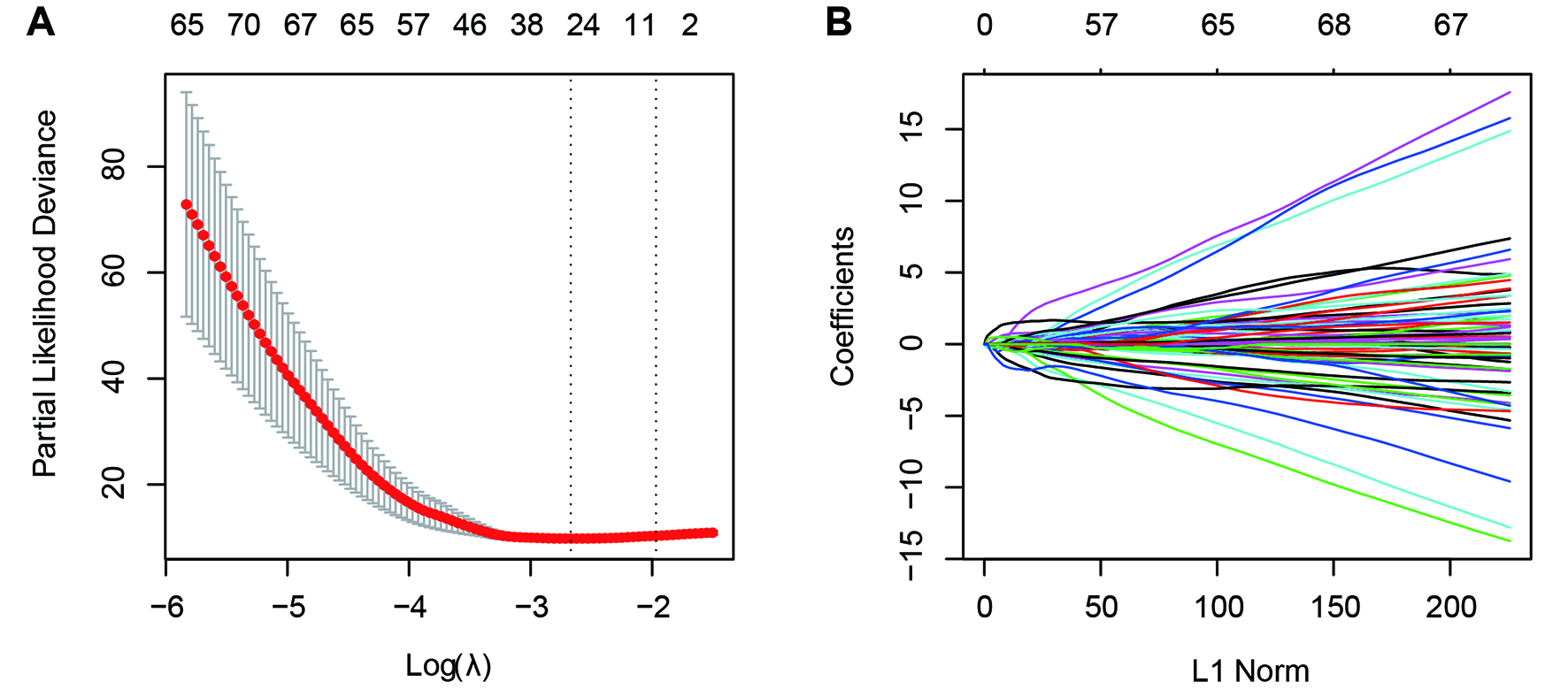

Supplement: Supplementary file 3 [file Image_2.TIF]

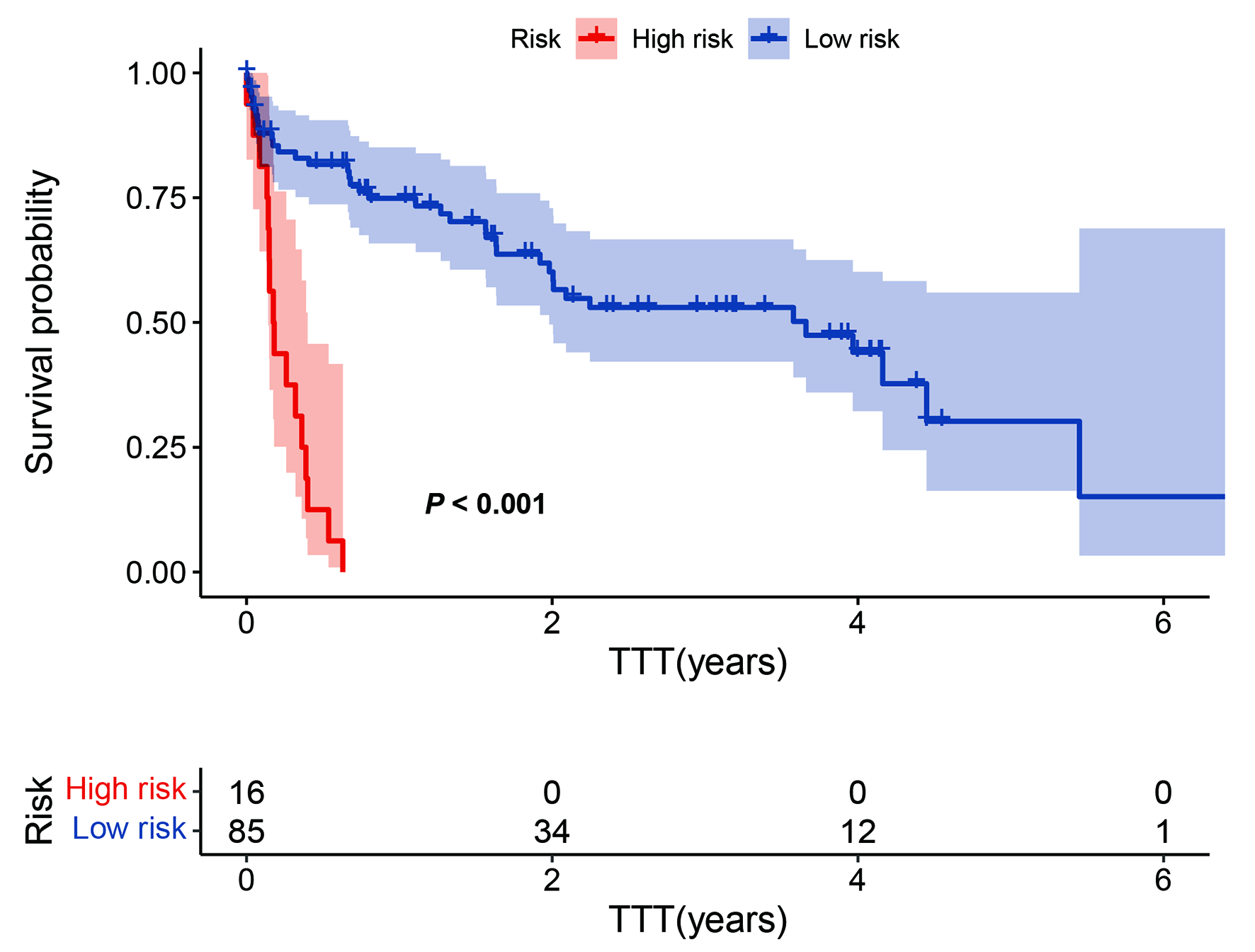

Supplement: Supplementary file 4 [file Image_3.TIF]

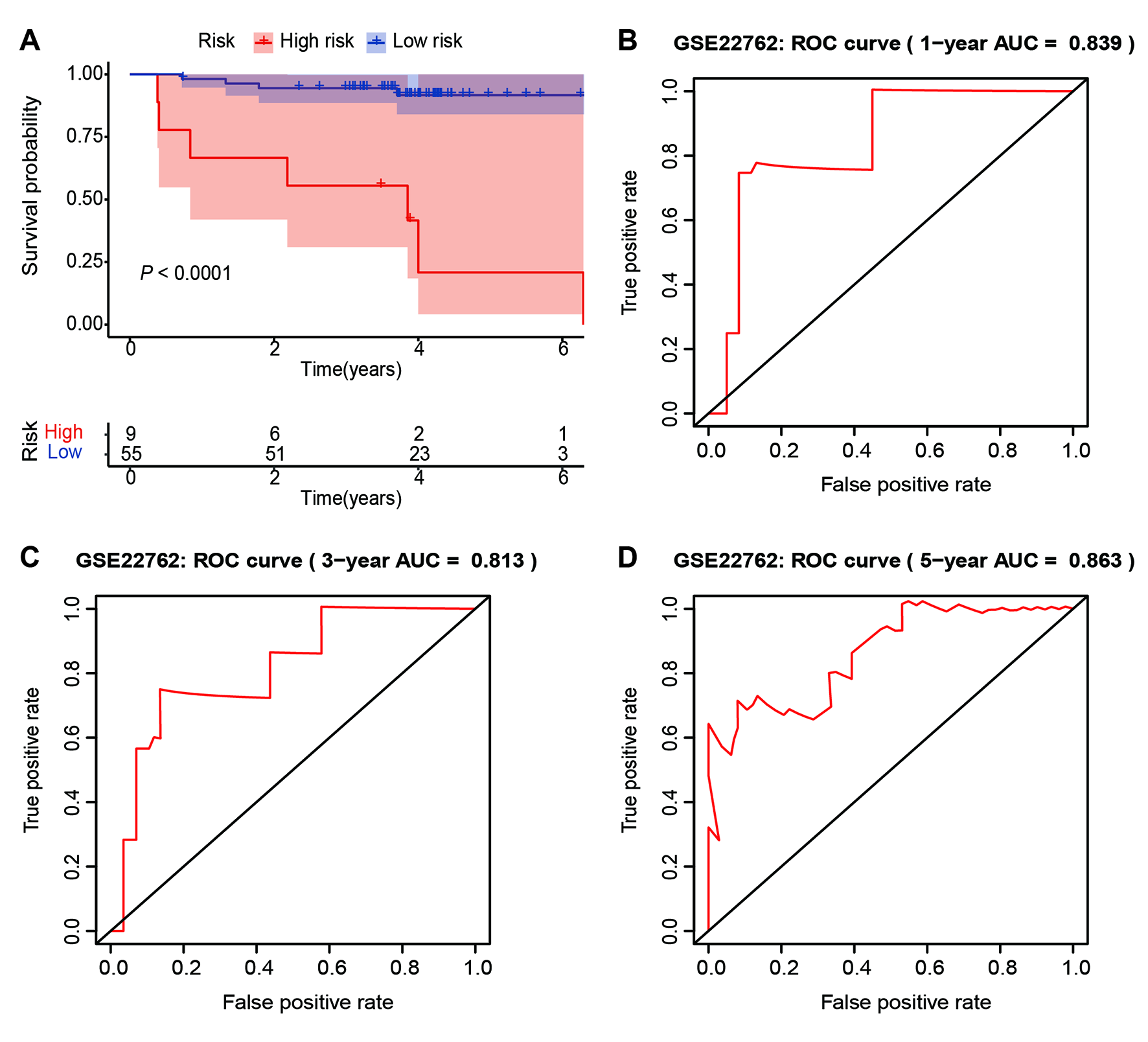

Supplement: Supplementary file 5 [file Image_4.TIF]
